# Therapeutic Uses and Efficacy of Botulinum Toxin in Orofacial Medicine: A Dental Perspective

**DOI:** 10.3390/toxins16050220

**Published:** 2024-05-10

**Authors:** Kazuya Yoshida, Merete Bakke

**Affiliations:** 1 Department of Oral and Maxillofacial Surgery, National Hospital Organization, Kyoto Medical Center, 1-1 Mukaihata-cho, Fukakusa, Fushimi-ku, Kyoto 612-8555, Japan; 2 Clinical Oral Physiology, Department of Odontology, Faculty of Health and Medical Sciences, University of Copenhagen, DK-2200 Copenhagen, Denmark; mbak@sund.ku.dk

Botulinum neurotoxin (BoNT) is the exotoxin of *Clostridium botulinum*, a Gram-positive, spore-forming bacterium. Kerner was the first to describe botulism [1] and Van Ermengem isolated the microorganism *Bacillus Botulinus* [2] but it was not until 1979 that Scott was able to make the first use of BoNT therapeutically for strabismus via injection to the extraocular muscles [3]. The clinical applications of BoNT have since dramatically expanded to treat ophthalmic, neurological, gastrointestinal, urological, orthopedic, dermatological, dental, secretory, painful, and other diseases [4,5]. Moreover, applications to the orofacial region have gained particular attention [6], targeting disorders such as oromandibular dystonia (OMD), hemifacial spasm, facial synkinesis, orolingual dyskinesia, functional dystonia, hemimasticatory spasms, trigeminal neuralgia, orofacial pain, temporomandibular disorders, temporomandibular joint dislocation, bruxism, palatal tremor, hypersalivation, spasmodic dysphonia, essential voice tremor, first-bite syndrome, and Frey syndrome [6]. In most cases the temporal and the masseter muscles are injected bilaterally as standard; however, treatment effects may be hampered as dystonia, spasms, and dyskinesia with involuntary jaw and tongue movements may be misdiagnosed due to the complexity of muscle activity and the involvement of several small muscles [7].

This Special Issue is focused on the use of BoNT in the orofacial region, from a dental perspective. Five papers reported on pain-related disorders, with three of these focusing on myofascial pain [Contributions 1–3], one on trigeminal or post-herpetic neuralgia [Contribution 4], and one on primary headache [Contribution 5]. Another four papers focused on OMD or neurological conditions [Contributions 6–8], and we also have a narrative review on the cosmetic applications of BoNT [Contribution 9].

BoNT alleviates muscle tension and pain, which is why the masticatory muscle pain associated with temporomandibular disorders or bruxism seems to be the area of greatest interest among dentists and oral surgeons [8]. De la Torre Canales et al. [Contribution 1] conducted a randomized controlled trial on BoNT-A and its effects on mandibular range of motion (pain-free, maximum unassisted and assisted mouth opening, and right and left lateral mandibular movements) and muscle tenderness in 80 patients with persistent myofascial pain. The patients were randomly divided into four groups (*n* = 20): three BoNT-A groups with different doses and a saline solution control group. The examination was performed blindly and according to the RDC/TMD classification [9]. After 28 and 180 days of treatment, compared with the control group palpation pain over the masseter and temporalis muscles were significantly reduced in all three BoNT-A groups regardless of dose. They concluded that BoNT-A improved mandibular range of motion and muscle tenderness in patients with persistent myofascial pain and that the effect was dosage independent. De la Torre Canales et al. [Contribution 2] also assessed the long-term effects of BoNT-A for 72 months in terms of subjective pain (VAS), pain sensibility (PPT), and muscle thickness (ultrasonography) in 14 patients with the same malady. They concluded that a single injection of BoNT-A provided long-term pain reduction in patients with persistent myofascial pain, and a reversibility of the negative effect on masticatory muscle thickness. Sitnikova et al. [Contribution 3] conducted a randomized clinical trial of BoNT injection in 57 patients with myalgia, myofascial pain, or headache attributable according to the Diagnostic Criteria for Temporomandibular Disorders (DC/TMD) and considered the degree and duration of the impairment of muscle performance after BoNT injection. The patients were randomly divided into two groups: one of which received BoNT-A first (*n* = 28) while the other received saline first (*n* = 29), with a cross-over timetabled in week 16, and a total follow-up period of 32 weeks. In total, 50 units were used in each patient with 2/3 bilaterally in the masseter and 1/3 in the temporalis. A significant reduction in electromyographic activity was observed up to week 18 *(p* ≤ 0.001), with full recovery at week 33. A significant reduction in the maximum bite force was observed up to week 11 (*p* ≤ 0.005), with full recovery at week 25. They conclude that muscle function recovery occurred within 33 weeks after BoNT-A, and thus considered that to be the period after which reinjections would be safe. The management of neuropathic pain is also challenging, and patients with the condition are often under the care of a dentist or an oral surgeon. Val et al. [Contribution 4] provided a systematic review regarding the clinical application of BoNT in managing neuropathic pain in the orofacial region, in which they analyzed five randomized clinical trials on classical trigeminal neuralgia, and one trial on post-herpetic neuralgia. The review concluded that the evidence supports the efficacy of BoNT injections for orofacial neuropathic pain management and the authors suggested that study protocols should be improved by considering patient selection, phenotyping, injection techniques, dosing, intervals between doses, etc. Moreover, an increased homogeneity among the research protocols is required to provide a protocol for the tailored use of BoNT for different orofacial neuropathic pain conditions.

Primary headaches, otherwise termed idiopathic headaches, are a large group of diseases, the etiology of the primary headache is unclear, and it is not a symptom of another known disease. The most common of these are migraines, tension-type headaches, and cluster headaches. Kępczyńska and Domitrz conducted a narrative review on the use of BoNT in chronic migraine and other primary headaches [Contribution 5], finding that BoNT is effective in pain control through its interaction with the soluble NSF attachment protein receptor complex, which inhibits the release of neurotransmitters, such as glutamate, substance P, and calcitonin gene-related peptide. BoNT-A is effective not only in reducing headache frequency and pain intensity, but also in improving other parameters including sufferers’ quality of life. They emphasize that many patients with these headaches seek dental care because orofacial pain is a common symptom; hence, an orofacial pain specialist should know about the diagnostic criteria for various types of headaches. Likewise, headache physicians should be aware of the semiologic aspects of orofacial pain.

Involuntary movements, such as dystonia, were the main target diseases when BoNT was first applied clinically [4,5]. OMD is a focal dystonia involving the masticatory, lingual, and/or muscles in the stomatognathic system [6], and symptoms related to OMD include masticatory disturbances, limited mouth opening, muscle pain, dysphagia, dysarthria, esthetic problems, and temporomandibular joint dislocation [6,10]. Bakke [Contribution 6] provided a review that introduced BoNT as a medical treatment for conditions of the orofacial region and focused on OMD and sialorrhea after supplementary training and according to the national guidelines, as these are relevant to dentists and also emphasized the need for interdisciplinary collaboration between dentists, neurologists and/or otologists for precise diagnostics and treatment. To achieve the best results and minimize or prevent side effects, standard doses should be used. Moreover, guidance on BoNT injections with electromyography or ultrasonography should be considered and regular and standardized treatments and controls should be investigated in future [Contribution 6]. The symptoms of OMD, such as jaw closing, jaw opening, and tongue dystonia, vary considerably according to the subtypes. In a follow-up questionnaire 8–10 years after the start of repeated BoNT treatment, which was answered by 14 persons with OMD, nine were still being treated with BoNT injections 3–4 times per year as monotherapy or combined with oral medication [7]. The most often reported dystonic symptoms were jaw closing and opening, as well as munching and chewing movements. In particular, eating and mental stress were reported to increase the dystonic activity, and sleep, relaxation, and quietness to decrease it. Most of the respondents felt that the BoNT treatment relieved their symptoms and the change of score a VAS scale for OMD symptoms was significant, with a reduction of 16%, either as an effect of the repeated BoNT injections or due to the time course [7]. Yoshida [Contribution 7] evaluated 408 patients [jaw closing dystonia, *n* = 223; tongue (lingual) dystonia, *n* = 86; jaw opening dystonia, *n* = 50; jaw deviation dystonia, *n* = 23; jaw protrusion dystonia, *n* = 13; and lip (labial) dystonia, *n* = 13] at baseline and after the end of BoNT therapy or in a stable status. All examiner-rated subscales (severity, disability, and pain) and patient-rated questionnaire scores (general, eating, speech, cosmetic, social/family life, sleep, annoyance, mood, and psychosocial function) were significantly lower at the endpoint than at baseline (*p* < 0.001). BoNT injection positively impacted the health-related quality of life, and the rating scale could evaluate both motor phenomena and non-motor symptoms when properly diagnosed and administered precisely. The rating scale can be used to comprehensively evaluate both therapeutic effects and health-related quality of life. Bilateral brain stimulation has been applied for the treatment of OMD, yet, for some patients, the symptoms do not improve sufficiently and additional BoNT injections may be required.

In clinical dentistry or oral surgery, we often encounter patients with orolingual dyskinesia or tardive syndrome. Skarmeta et al. [Contribution 8] reported a rare case with Trazodone, a (tetracyclic antidepressant)-induced OMD. Early identification and assessment of tardive symptoms are imperative for successful treatment, and Trazodone should be prescribed with caution in patients taking other medications with the potential to cause tardive syndromes. Preventing and identifying the onset of tardive syndromes is of paramount importance, and the authors suggested that clinicians should balance the risk/benefit ratio before prescribing drugs that can potentially produce tardive symptoms.

The cosmetic use of BoNT in clinical practice is significant. It is an important and interesting field for many clinicians and patients and there has been a global rise in the demand for non-invasive procedures to help patients rejuvenate their appearance. BoNT is now the most performed esthetic procedure in the world and various types of BoNT with different properties have become available. Hong provided an excellent practical illustrative guide and reference for BoNT applications in the orofacial area including safe injection areas and effective doses [Contribution 9]. To ensure safety and effectiveness, appropriate guidelines on the use of BoNT injections for the treatment of dynamic rhytides and hypertrophic structure is warranted.

This Special Issue is representative of dental professionals who use BoNT injections worldwide, with contributors who are specialists in the relevant fields, producing papers that examined the treatment’s effectiveness and safety. However, the approved indications for BoNT injections may vary between countries and, to be officially approved for a certain indication, evidence from well-designed, randomized, controlled trials with larger sample sizes and longer follow-up periods are essential. If the number of experts in each country is limited, international collaborative research may be necessary. To facilitate the use of BoNT widely and routinely by general practitioners guidelines for safe and effective treatment must be established.

BoNT is very expensive, meaning that there is a possibility that some clinicians will overlook conventional treatment methods and use BoNT instead for profit. Consequently, the medical costs may skyrocket. Therefore, supplementary training or some kind of qualification should be the basic requirement for using BoNT therapeutically. In addition, differential diagnosis is important for neurological diseases, such as OMD, and collaboration between neurological and dental experts is advisable [Contributions 5–7]. Since dental professionals are specialists of the stomatognathic system, they, rather than medical professionals, may have the skills to provide more accurate injections to target the muscles of interest in the oral region. Ultimately, effective and safe BoNT therapy requires a multidisciplinary team approach.
